# Metals and Metallothionein Expression in Relation to Progression of Chronic Kidney Disease of Unknown Etiology (CKDu) in Sri Lanka

**DOI:** 10.3390/diseases10020034

**Published:** 2022-06-12

**Authors:** S. H. Nandana P. Gunawickrama, A. Rajith N. Silva, P. G. Chandra L. Nanayakkara, K. B. Suneetha Gunawickrama, J. M. Kithsiri B. Jayasekara, Naduviladath V. Chandrasekharan

**Affiliations:** 1 Institute for Combinatorial Advance Research and Education, General Sir John Kotelawala Defence University, Ratmalana 10390, Sri Lanka; 2 Department of Basic Sciences, Faculty of Allied Health Sciences, General Sir John Kotelawala Defence University, Boralesgamuwa 10290, Sri Lanka; nsrajith@kdu.ac.lk; 3 Department of Anatomy, Faculty of Medicine, University of Ruhuna, Galle 80000, Sri Lanka; pgclnana@med.ruh.ac.lk; 4 Department of Zoology, Faculty of Science, University of Ruhuna, Matara 81000, Sri Lanka; suneetha@zoo.ruh.ac.lk; 5 Department of Medical Laboratory Sciences, Faculty of Allied Health Sciences, General Sir John Kotelawala Defence University, Boralesgamuwa 10290, Sri Lanka; kbjayasekara@kdu.ac.lk; 6 Department of Chemistry, Faculty of Science, University of Colombo, Colombo 00300, Sri Lanka; nchandra@chem.cmb.ac.lk

**Keywords:** chronic kidney disease of unknown etiology (CKDu) in Sri Lanka, CKD, proteinuria, eGFR, UACR, metallothionein 1A, metallothionein 2A, metals

## Abstract

Chronic kidney disease of unknown etiology was investigated for metal relations in an endemic area by a cross-sectional study with CKD stages G1, G2, G3a, G3b, G4, G5 (ESRD), and endemic and nonendemic controls (EC and NEC) as groups. Subjects with the medical diagnosis were classified into groups by eGFR (SCr, CKD-EPI) and UACR of the study. It determined 24 metals/metalloids in plasma (ICPMS) and metallothionein (MT) mRNA in blood (RT-PCR). MT1A at G3b and MT2A throughout G2–G5 showed increased transcription compared to NEC (ANOVA, *p* < 0.01). Both MT1A and MT2A remained metal-responsive as associations emerged between MT2A and human MT inducer Cr (in EC: r = 0.54, *p* < 0.05, n = 14), and between MT1A and MT2A (in EC pooled with G1–G5: r = 0.58, *p* < 0.001, n = 110). Human MT (hMT)-inducers, namely Zn, Cu, As, Pb, and Ni; Σ hMT-inducers; 14 more non-inducer metals; and Σ MT-binding metals remained higher (*p* < 0.05) in EC as compared to NEC. Declining eGFR or CKD progression increased the burden of Be, Mg, Al, V, Co, Ni, Rb, Cs, Ba, Mn, Zn, Sr, Σ hMT-inducers, and Σ MT-binding metals in plasma, suggesting an MT role in the disease. MT1A/2A mRNA followed UACR (PCA, Dendrogram: similarity, 57.7%). The study provides evidence that proteinuric chronic renal failure may increase plasma metal levels where blood MT2A could be a marker.

## 1. Introduction

The chronic kidney disease of unknown etiology (CKDu) in Sri Lanka is endemic nephropathy with distinct geographical distribution. The disease remains enigmatic as medically recognized risk factors of chronic kidney disease [1,2] do not justify its high prevalence in the north-central province of the country. CKDu progresses via tubulointerstitial damage [3] that culminates in end-stage renal disease (ESRD). In chronic kidney disease (CKD), the damage to tubulointerstitium may be initiated by reactive oxygen species (ROS) generated due to nephrotoxins such as increased metals [4] and reabsorbed albumin during albuminuria [5] in tubular cells. In this context, exogenous exposure to trace and heavy metals were postulated as causal to chronic renal failure in the region. However, the literature remains inconclusive as chemical factors such as groundwater fluorides [6], Cd [7], dietary Cd [8], and As [9] have been suggested, with negation over many metals and metalloids such as As, Cd, Pb, Fe, Zn, Mn, Cr, Co, Ni, Cu, Mo, Ag, Hg, and U [10,11,12].

Metallothioneins (MT) are single-polypeptide proteins that sequester and detoxify a variety of metals utilizing its thiol side chains of twenty Cysteine residues in structure. MT includes metal-inducible MT1A and MT2A genes whose tissue distribution is ubiquitous in rodents and man [13]. The known induction mechanism qualifies them as markers of metal exposure. Inducers of human metallothioneins (hMT) include Cd, Zn, Cu, Cr [14], Pb, As [14,15], Ni [14,16], Hg, and Bi [17]. Basal expression of MT1A/2A occurs when metal-activated transcription factor-1 (MTF-1) binds to metal response elements (MRE) in the upstream promoter region of the gene [18]. MTF-1 assumes MRE binding conformation by dissociation from its dimerization partner, metal transcription inhibitor (MTI), in a Zn-dependent manner. The metal inducers, in fact, operate by influencing Zn availability [19]. In its functions, MT in both intra- and extracellular environments binds Cu, Ag, Zn, Cd, Co, Ni, Fe, Pb, Bi, Hg, Au, Pt, and As [20] selectively in valence-dependent coordination with Cys-SH groups. The binding constitutes a buffering activity that detoxifies excess metals and attenuates metal-rendered oxidative stress.

Chronic kidney disease (CKD) associates an inflammatory process [21] marked by inflammatory cytokines such as blood MCP-1 and IL-6 [22] and C-reactive protein to albumin ratio in serum [23]. Metallothioneins are involved in the pathology of inflammatory diseases [24] and in the prevention of diabetic nephropathy [25,26] in particular. A correlation was shown between MT induction and renal dysfunction in human subjects [27], so the hypothesization of interrelation among metals, MT expression, and chronic renal damage is reasonable.

In such backdrop, the study followed blood MT1A/2A mRNA expression and plasma levels of twenty-four metals and metalloids throughout the entire length of disease progression in subjects from CKDu endemic Padaviya (PDV) area. Results were expected to be useful in the initial identification of the pathways leading to the high-prevalence chronic renal failure in the area.

## 2. Materials and Methods

**Scheme 1:**
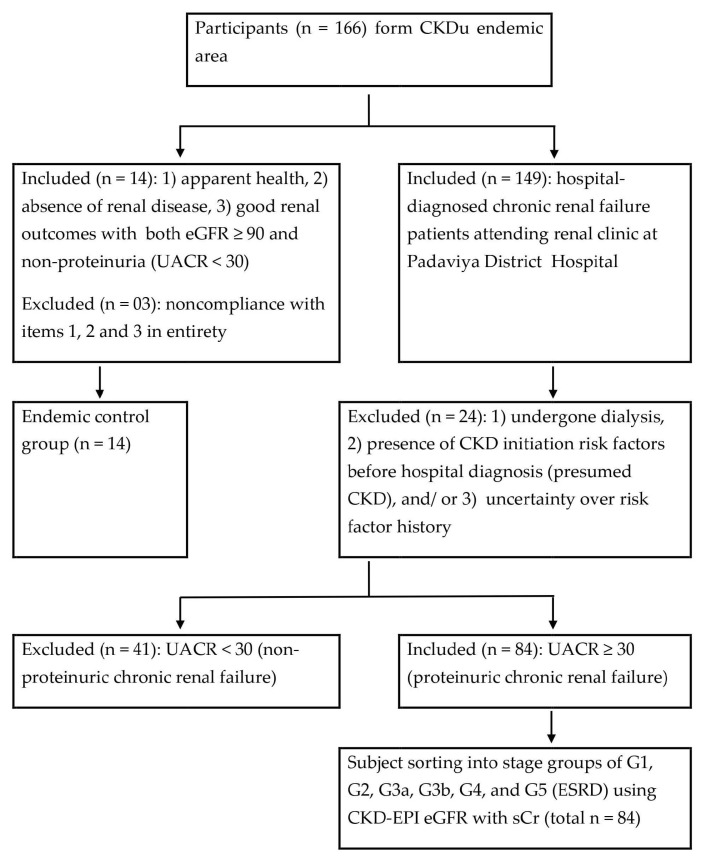
Subject classification into study groups. eGFR: estimated glomerular filtration rate (mL/min/1.73 m^2^); UACR: urine albumin to creatinine ratio (mg/g); G1–G5: CKD development stages; ESRD: end-stage renal disease (i.e., G5). The study utilized its own eGFR and UACR determinations. Normoalbuminuric subjects (n = 41) were excluded as their renal status was unconfirmed in the study. The nonendemic control group (included n = 12, excluded n = 13) was constituted using the same criteria as for the endemic control.

A cross-sectional study was conducted in CKDu endemic Padaviya (PDV) area with volunteer participation of subjects following informed consent (Scheme 1). The recruited were already diagnosed with chronic renal failure (CRF) and attending the pre-dialysis renal clinic at PDV district hospital. The study comprised only males as gender influences metallothionein expression in mammals [28,29] and humans [30]. An endemic control (EC) from PDV general area and a nonendemic control (NEC) from Padalangala (PDL) were also established. PDV and PDL are non-contiguous and comparable in climate, sub-culture, and socioeconomics. A total number of 178 subjects (age range, 36–79 years) participated in the study.

### 2.1. Sample Collection and Initial Processing

Subsequent to a questionnaire on demography, whole blood and spot urine was obtained from each subject. About 300 µL EDTA whole blood was immediately added into 1.3 mL RNA-later (AM1928, Ribopure™ Blood Kit, Life Technologies, Carlsbad, CA, USA) in a 2 mL RNAse free reaction vial, mixed gently, and kept at room temperature for 20 min. Plasma and serum were obtained from EDTA, added, and clotted the whole blood, respectively (1500 g × 10 min). After processing, RNA-later-added EDTA-blood, plasma, serum, and urine aliquots were kept on dry ice for transportation to the laboratory before storing at −80 °C on the same day.

### 2.2. Kidney Dysfunction Markers

Serum creatinine (SCr, mg/dL), urine albumin (UA, mg/L), and urine creatinine (UCr, mg/dL) were measured (automated chemistry analyzer, Mindray BS-200, Shenzhen, China) by enzymatic colorimetric, immune-turbidimetric, and Jaffe assays, respectively. Serum cystatin C (SCysC, mg/L) was measured (automated protein analysis system, QR100-Heales, Shenzhen, China) by latex-enhanced turbidimetric assay. All determinations were performed under diagnostic quality control (QC) standards of the equipment/reagent manufacturer at a clinical laboratory.

The estimated glomerular filtration rate (eGFR, mL/min/1.73 m^2^) was calculated by the chronic kidney disease epidemiology collaboration (CKD-EPI) equation using SCr [30]. Urine albumin to creatinine ratio (UACR, mg/g) was derived from urinary albumin (mg/L) and creatinine (mg/dL) levels. The eGFR was also estimated by modification of diet in renal disease (MDRD) by SCr [2], CKD-EPI by Cystatin C, and CKD-EPI by SCr and Cystatin C equations [31].

### 2.3. Subject Classification

Subjects were sorted into the CKD stages and control groups considering both eGFR (CKD EPI, SCr) and UACR, as proposed by Stevens and Levin [32]. Briefly, individuals with good renal outcomes (both eGFR ≥ 90 and UACR ≤ 29) and no hospital diagnosis of renal disease were recruited to EC and NEC. Subjects were tentatively assigned CKD progression stages, G1, G2, G3a, G3b, G4, and G5 (end-stage renal disease, ESRD) when they were within eGFR ranges of >89, 89–60, 59–45, 44–30, 29–15, and <15, respectively. In each case, G1 to G5 inclusion is required to be albuminuric (UACR ≥ 30). Subjects with discrepancies were excluded as their renal status was inconclusive.

### 2.4. Metallothionein Expression

MT1A and MT2A mRNA levels were determined by reverse transcription-polymerase chain reaction (RT-PCR). RNA preservation in EDTA whole blood and extraction of total RNA were performed using RiboPure™ Blood Kit (AM1928). Total RNA kept at −20 °C was subsequently reverse transcribed in a reaction mixture that comprised Moloney Murine Leukemia Virus Reverse Transcriptase (M-MLV RT), dNTPs, Ribonuclease inhibitor, and reaction buffer (pH 8.3, 250 mM Tris-HCl, 375 mM KCl, 15 mM MgCl2, and 50 mM DTT). Random hexamer primer and poly dT primer were added with supplementary nuclease-free (NF) water to a final volume of 25 μL. Vials were incubated at 44 °C for 1 h followed by 94 °C for 10 min to inactivate RT activity before storing at −20 °C for forthcoming qPCR. cDNA equivalent to 80 ng of total RNA was used for separate PCR amplification of MT1A and MT2A amplicons using specific primer pairs reported before [33]. Thermal cycling commenced with an initial melting at 95 °C for 5 min. Optimized reaction conditions spanned a maximum of 40 cycles for both MT1A and MT2A with annealing at 51 °C and 53 °C, respectively, elongation at 72 °C, and melting at 94 °C. Each step was held for 30 s, with the final elongation kept over for 50 s. Concurrent negative controls were run, substituting NF water for M-MLV RT in the previous step and for cDNA yield to verify amplification. The housekeeping glyceraldehyde-3-phosphate dehydrogenase (G3PDH) gene was co-amplified according to Abdel-Mageed and Agrawal [34] to confirm the template quality of the PCR step. The real-time cycle threshold (Ct) was taken as the reaction end-point.

Post-amplification PCR mixtures were verified for products by electrophoresis in 1% agarose gel containing ethidium bromide, with a DNA ladder in one lane. UV illuminated gel was visualized and analyzed by densitometric scanning (Bio-Rad GS-700 Imaging Densitometer, Hercules, CA, USA) using appropriate software (Bio-Rad CFX Manager 3.1).

### 2.5. Plasma Metal Levels

Blood plasma (1 mL) was analyzed for twenty-four trace metals and metalloids by inductively coupled plasma mass spectrometry (7900 ICP-MS with ASX-500 Series ICP-MS auto sampler, Agilent Technologies, Santa Clara, CA, USA). Glassware kept in 10% HNO_3_ over 48 h and washed thoroughly with deionized water was used in the protocol. Initially, 10 mL 70% HNO_3_ was added to 1 mL plasma. It was followed by microwave acid digestion over 20 min and then the addition of 50 mL of deionized water to have the final acidity at 14%. The mixture was filtered into a capped plastic tube. HNO_3_ utilized was of analytical grade, and the blanks were prepared similarly with deionized water substituted for plasma. Agilent multi-element standard solution 2A was serially diluted to obtain 5, 20, 50, 80, 100, 250, 500, and 1000 ppb solutions as standards. Cu, As, and V were measured in He gas mode, whereas other elements were assayed in no-gas mode. Data were normalized to total plasma protein (mg/mL) in order to exclude variation due to differential manual practice. Total protein was measured by the Bradford method [35] using bovine serum albumin as the standard.

### 2.6. Statistical Analyses

Demographic factors in association with CRF prevalence were identified by estimating Odds Ratio (OR). Means of MT1A mRNA, MT2A mRNA, and plasma metal levels among EC, NEC, and stages G1 through G5 were compared by one-way ANOVA followed by the post hoc test, Tukey HSD. Pearson’s correlation assessed linear associations involving MT1A/2A, plasma metal levels, and kidney dysfunction markers. Nonlinear relations were revealed with principal component analysis (PCA) by correlation-based matrix and with cluster dendrogram involving complete linkage and correlation coefficient distance. Metal species that showed a stronger correlation coefficient than ±0.2 (Pearson’s test) with any of MT or kidney dysfunction marker variables were included in the multivariate analyses. Statistical procedures and plotting were conducted with Minitab^®^ 17.1.0 software.

## 3. Results

Proteinuric CRF subjects from the endemic area comprised 89% of rice-growing field farmers (Table 1). Likelihood of the disease development (OR > 1) was significant with field farming (*p* < 0.001) and use of agrochemicals (*p* < 0.05).

**Figure 1:**
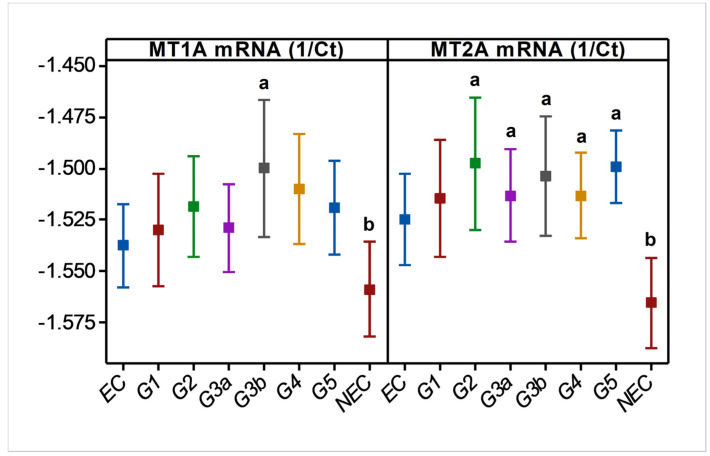
Variation in blood MT1A and MT2A mRNA levels during CRF progression in CKDu endemic area. MT; metallothionein, Ct; cycle threshold (RTPCR), EC; endemic control, and G1-G5; CKD stage groups, NEC; nonendemic control. n = 110. Log10 MT data are shown as mean and 95% confidence interval in linear scale Y axes. a and b indicate significance at *p* < 0.01 between two means (one-way ANOVA, Tukey HSD).

**Figure 2:**
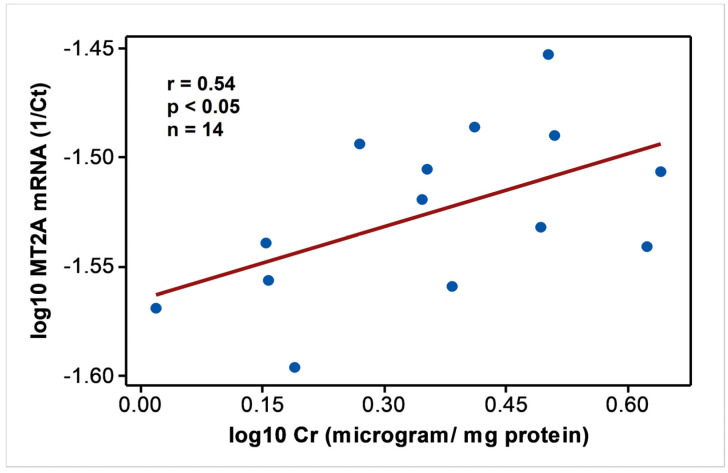
MT2A mRNA expression follows hMT inducer; Cr in individuals with good renal outcomes in CKDu endemic Padaviya area. Data were tested for Pearson’s correlation and plotted in linear scale axes.

**Figure 3:**
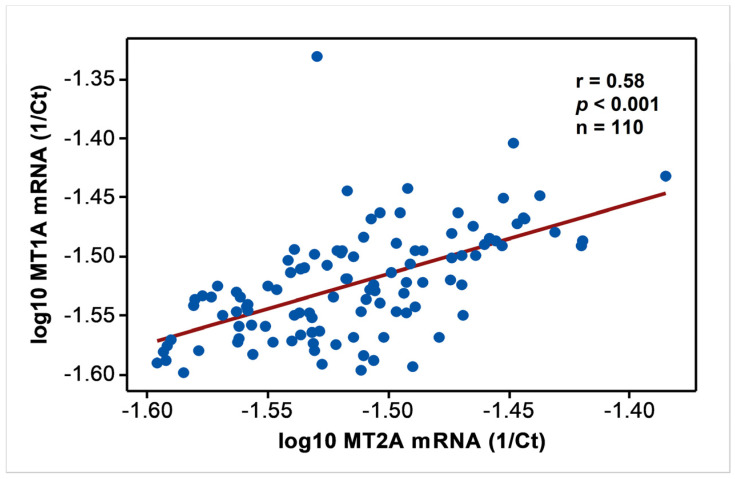
Comparable expression of MT1A and MT2A mRNA in the study. Data from endemic control, nonendemic control, and CKD stages G1–G5 were tested for Pearson’s correlation and shown in linear scale axes.

In endemic PDV areas, MT1A and MT2A transcript levels between EC and CKD groups (Figure 1) were not different (*p* > 0.05). In comparison to NEC, however, both genes showed upregulation (*p* < 0.01) as disease development took place. MT2A was more responsive, and all CKD groups from G2 to G5 had significantly high expression levels (*p* < 0.01) compared to NEC. Metal inducibility of MT was evident in EC (Figure 2) as hMT inducer Cr in plasma was in positive correlation with blood MT2A mRNA (r = 0.58, *p* < 0.05, n = 14). Similarly, MT1A and MT2A mRNA expressions remained directly proportional (r = 0.58, *p* < 0.001, n = 110), pointing to comparable gene expression regulation mechanisms and shared inducers between the two (Figure 3).

Comparison between NEC and EC showed that plasma content of many metals and metalloids was significantly higher in the endemic area in individuals with good renal health (Table 2).

**Figure 4:**
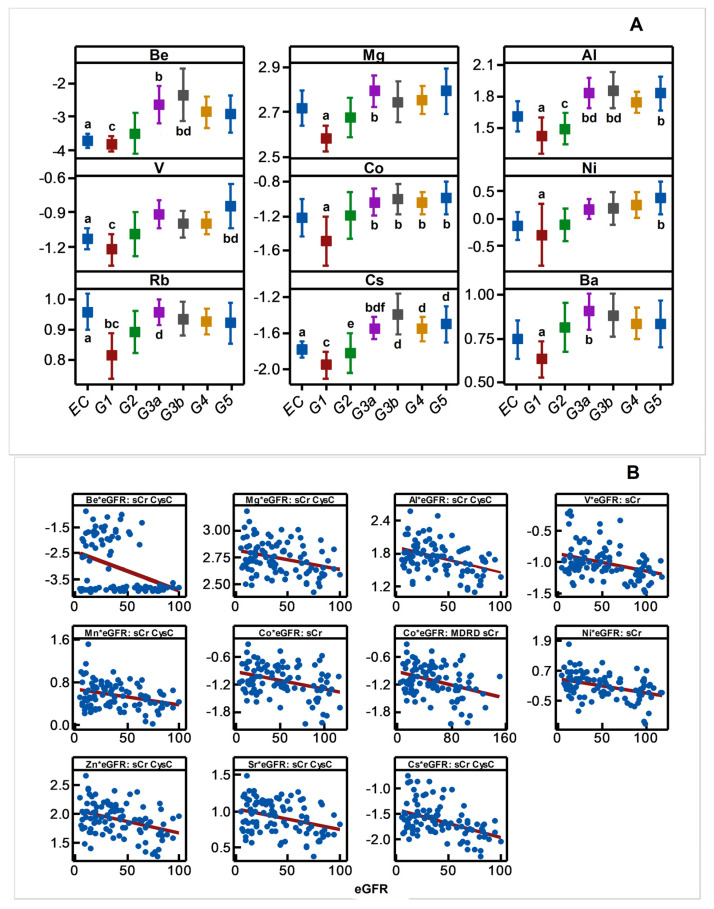

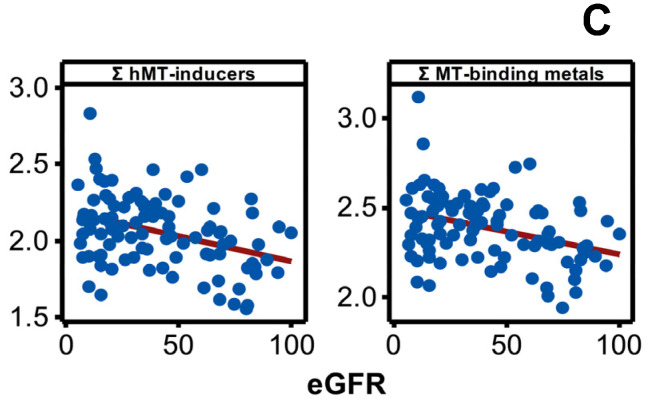
Variation in plasma metal levels as chronic renal failure progresses in CKDu endemic Padaviya area. Log10 transformed plasma metal levels (µg/mg protein, by ICPMS) were tested for (**A**) statistical significance among study groups (EC: endemic control, G1-G5: CKD stages, n = 97−98) by one way ANOVA/Tukey HSD. Data represent mean and 95% confidence interval. Letter pairs a and b, c and d, and e and f indicate statistical difference (*p* < 0.05). Data of unaffected metals and metalloids are not shown. In Figure (**B**,**C**), associations with eGFR (mL/min/1.73 m^2^) variants as kidney dysfunction markers by Pearson’s test are shown. Metals with rho weaker than ±0.3 were not considered, and the strongest correlation by others is shown. In Figure (**B**), eGFR are CKD-EPI except for Co against MDRD. Figure (**C**) plots eGFR of CKD-EPI (sCr, CysC). All Y-axes and X-axes of Figure (**B**,**C**) are on linear scale.

Disease progression altered the plasma concentration of Be, Mg, Al, V, Co, Ni, Rb, Cs, Ba, Mn, Zn, and Sr, and it included increases (*p* < 0.05) in the plasma content of many metals and metalloid species towards middle or advanced stages of the disease progression (Figure 4A). The result was consistent with negative associations shown by plasma Be, Mg, Al, V, Co, Ni, Cs, Mn, Zn, Sr, Mn, Zn, and Sr with eGFR spanning the entire range of disease progression (Figure 4B) revealing an increasing plasma metal burden as the kidney dysfunction aggravates. The same trend emerged when metals were selectively grouped and considered as total hMT inducers and total MT-binding metals (Figure 4C).

**Figure 5:**
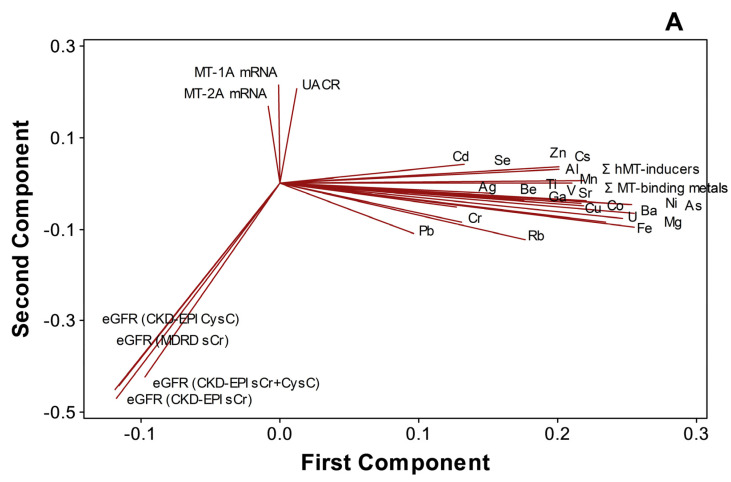

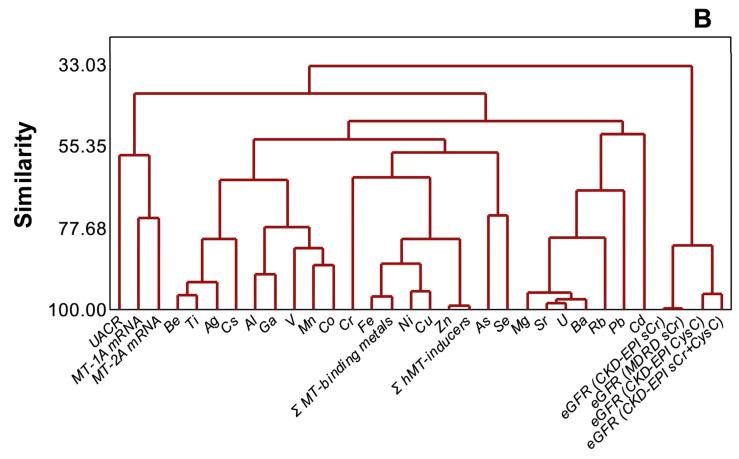
Distinct clustering of plasma metals, MT1A/2A transcripts, and kidney dysfunction markers in CKDu endemic Padaviya area (n = 97), (**A**) principal component analysis, (**B**) cluster dendrogram.

PCA (Figure 5A) and cluster dendrogram (Figure 5B) projected that metals and metalloids were not necessarily related to either kidney dysfunction markers or MT1A/2A expression during disease progression (n = 97). In the analyses, MT1A mRNA and MT2A mRNA (similarity, 74.8%), MT1A/2A mRNA and UACR (similarity, 57.7%), and metals and metalloids (similarity, 48.4%), as well as eGFR variants (similarity, 82.3%), were separately clustered. Total MT-binding metals remained closely related to Fe (similarity, 96.38%) and Cu (similarity, 87.27%), as total MT-inducers had a similarity of 99.03% to Zn in the endemic area.

## 4. Discussion

CKD cases are prevalent in the CKDu endemic areas in the north-central province of Sri Lanka [36,37], and CKD may be distinguished by initiation risk factors (IRF) [1,2] that existed prior to diagnosis with poor renal outcomes. Identification of CKDu cases with certainty involves ambiguity in field centers as risk factor history obtained from subjects may not be totally dependable. Local medical practices do not discriminate between the two, as symptoms and management appear to be similar. Despite the fact that CRF subjects with a clear history of IRF were excluded (Scheme 1), the groups G1–G5 in the study may comprise CKD and CKDu cases due to under-reporting. For this reason, the authors prefer the terminology chronic renal failure cases from CKDu endemic PDV area to describe the G1–G5 groups of the study.

The study classified participants into control and CKD stages [32] using spot eGFR (CKD-EPI, sCr) and UACR. The stage groups from G1 to G5 comprised proteinuric subjects as inclusion was based on obligatory UACR > 29 mg/g that confirmed renal failure [32]. EC and NEC were nonproteinuric (UACR ≤ 29) with eGFR ≥ 90. This approach permitted the following of the entire range of disease progression from good renal health (control) to CKD stages, G1 through G5 (ESRD), at one time-point in a cross-sectional study design.

Plasma metal levels are markers of metal exposure. Blood cell MT-1 and MT-2 transcripts were reported in rodents and humans as specific markers of metal exposure or effect [38]. Blood plasma harbor metals in free as well as protein-bound forms. The acid digestion performed prior to ICPMS determination would liberate all bound metals so that reported metal concentrations represent the total plasma content (burden) of individual elements each. MT1A and MT2A transcripts were measured in the study for verification of plasma metal levels and as metal-responsive markers in the same biological compartment.

### 4.1. Demography

OR analyses were consistent with prior reports [36,39] that the paddy farmers were the most affected occupational category (Table 1, *p* < 0.001) in PDV and adjacent endemic areas. Paddy farmers use agrochemicals as an occupational practice where the substance handling and application tend to be arbitrary, often unrestrained, and without safety gear. Identification of rice-growing field farmers as a vulnerable group to the disease and of agrochemical usage as a risk factor (OR > 1, *p* < 0.05) in the present study is notable.

High prevalence among farmers may show that the initiation of chronic renal failure in PDV may involve a hitherto unidentified etiologic factor or effect. The metals are impurities of agrochemicals as in the cases of As, Pb, and Cd in pesticides [40]; As, Co, Cr, Ni, and Pb in herbicides [41]; and Fe, Mn, Cu, Cd, and Cr in inorganic fertilizer in Sri Lanka [42]. This context suggests that paddy farmers risk metal exposure beyond background levels with substandard practices of agrochemical usage. However, evidence for an association between agrochemical usage and world CKDu epidemics is not conclusive [43]. As farming and agrochemical usage are inevitably associated, the data leading to the high OR of the latter could be incidental.

### 4.2. Plasma Metals and Metallothionein

Enhanced MT1A and MT2A mRNA levels in subjects with chronic kidney disease (*p* < 0.01, Figure 1A and Figure 2A) compared to NEC rather than EC, and higher metals and metalloid levels (*p* < 0.05) in EC compared to NEC (Table 2) suggest that elevated plasma burden of metals could be the reason for MT upregulation in an endemic area. Positive correlations between MT2A mRNA and hMT inducer, Cr in EC/PDV (Figure 2), and between MT1A and MT2A transcripts in all subjects/PDV (Figure 3) show metal responsiveness of MT2A and shared expression regulation between two genes, respectively. Higher plasma burdens of many metals and metalloids (Table 2) may suggest greater exogenous exposure to the elements in CKDu endemic areas. Notably, Cu, Pb, and As among them are hMT inducers [13], which could partly explain MT upregulation in the area.

Besides MRE-mediated induction by hMT inducer metals, excessive metals, in general, may shift intracellular redox status [4], either alone or in synergy, and enhance transcription via antioxidant response elements (ARE) in the upstream promoter region of the gene [44]. It is plausible that both MRE-dependent inducers and other metals may upregulate hMT under low and persistent exposures.

### 4.3. Plasma Metals and CKD Progression

Reports on tissue metal burden over CKD progress were scarce in the literature, posing a constraint in assessing the present results. However, where data are available, particularly from epidemiological approaches, statistical associations between plasma concentrations of metals and metalloids with declining eGFR were shown for elements including aluminum, arsenic, barium, lead, molybdenum, rubidium, strontium, vanadium, and zinc [45]. In general agreement with plasma trends, increasing kidney dysfunction was seen to reduce urinary metal excretion across the entire eGFR range [46]. In the present study, intergroup comparison of individual metal data (Figure 4A) and testing of linear correlation (Figure 4B), respectively, suggests disrupted homeostasis and metal accumulation in plasma as the chronic renal disease progresses in CKDu endemic PDV area. Involvement of the kidneys in metal homeostasis is known for Ni [47], Fe [48], Zn [49], Cu [50], As, Cd, Pb [51], Hg [52], Cs Cr, U [53], and Al [54]. It involves adequate filtration and reabsorption from tubular lumen [55] so that progressive tubulointerstitial damage in CKD/CKDu [3] should be fundamental to the observed variation. The sizable number of subjects with unaltered metals (*p* > 0.05) despite disease progression (Figure 4A,B) is intriguing. Perhaps, unaffected nephrons known for compensational hyperactivity [56] may be maintaining homeostasis to variable extents yet triggering further pathogenesis [56]. The results show that progression of the endemic chronic renal failure in the PDV area did not alter Li, Cr, Fe, Cu, Ga, As, Se, Ag, Cd, Ti, Pb, and U levels (*p* > 0.05 in ANOVA, r lesser than ±0.3, and *p* > 0.05 in Pearson test). Thus both affected and unaffected elements occur during the progressive renal damage.

Distinct clustering of individual metals, Σ hMT-inducers, and Σ MT-binding metals in plasma from eGFR variants and UACR (Figure 5A,B) is not consistent with a role of plasma metal burden as a risk factor of the chronic renal failure in the endemic area. Nevertheless, the high similarity of MT1A mRNA and MT2A mRNA with UACR points to MT upregulation in relation to the disease progression in the CKDu endemic PDV area. Gene expression of hMT1A/2A is regulated by stress, reactive oxygen species (ROS), inducer metals, and cytokine signaling via glucocorticoid response elements (GRE), ARE, MRE, and STAT (signal transducers and activators of transcription) in the promoter region, respectively. Increased plasma metal levels, hMT-inducers, and MT-binding metals in EC (Table 2) and plasma metal accumulation in relation to kidney dysfunction (Figure 4B,C) could alter redox status and generate ROS (reactive oxygen species), leading to ARE signaling. Increased oxidative stress and pro-inflammatory cytokines in CKD stage G3–G5 patients had been shown before [57]. Pro-inflammation in terms of spiked IL-6 and MCP-1 at G3b was reported in CRF patients in the CKDu endemic area of Sri Lanka [22]. Thus one or more pathways, alone or in concert, could upregulate metallothionein expression in the PDV area.

Exceeding similarity values (Figure 5B) between redox cycling Fe, Cu [20], and MT-binding metals and between Zn that facilitates MT induction by metals [19,20] and hMT-inducers suggest that MT expression remained responsive in subjects from the endemic area. Cr-upregulated MT2A (Figure 2) and associated mRNA expression levels between MT1A and MT2A (Figure 3) remain further evidence.

## 5. Conclusions

The study provided evidence that progression of proteinuric chronic renal failure in CKDu endemic areas increases plasma metal levels. MT2A gene expression could be a marker in such a context.

## Figures and Tables

**Table 1:** Paddy farmers were at risk of developing chronic renal failure (CRF).

	Endemic Control ^1^ (n, 14)	CRF Subjects ^1^ (n, 84)	Odds Ratio ^2^ (95% CI)	Non Endemic Control ^3^ (n, 12)
Gender/male	100	100	-	100
Age-range (year)	40–63	36–77	-	45–60
*Occupation and practice*				
Labourer	7.1	0	0.05 (0.002–1.3)	0
Paddy farmer	35.7	89.2	15.0 (4.1–54.6) **	91.6
Civil defence force/part time paddy farming	50	7.14	0.07 (0.02–0.29) **	8.3
Government employee	0	3.5	1.24 (0.06–25.4)	0
Unemployed	7.1	0	0.05 (0.002–1.3)	0
Agrochemical usage	78.5	96.4	7.36 (1.31–41.1) *	91.6

^1^ From CKDu endemic Padaviya, ^2^ between endemic control and CRF subjects, ^3^ from Padalangala. Data represent percentages from group total. * *p* < 0.05, ** *p* < 0.001 (Z distribution). *p* > 0.05 for education level reached and domestic water source categories (data not shown).

**Table 2:** Plasma metal and metalloid levels in subjects with good renal outcomes between CKDu endemic ^1^ and nonendemic ^2^ areas.

	EC ^1^ (µg/mg Protein)	NEC ^2^ (µg/mg Protein)	*p*
Li	0.86 ± 0.35	0.04 ± 0.003	***
Mg	547.8 ± 50.5	366.2 ± 16.7	**
Al	46.5 ± 6.27	16.9 ± 1.70	***
V #	79.2 ± 7.50	46.8 ± 5.70	**
Mn	3.08 ± 0.34	1.83 ± 0.14	**
Fe	126.2 ± 13.3	84.2 ± 6.69	**
Co	84.3 ± 20.5	19.1 ± 4.10	**
Ni	1.02 ± 0.19	0.35 ± 0.12	**
Cu	17.9 ± 1.02	13.4 ± 0.57	**
Zn	82.7 ± 17.6	33.4 ± 3.37	**
Ga #	0.99 ± 0.05	0.81 ± 0.02	**
As	1.37 ± 0.12	2.43 ± 0.21	***
Rb	9.37 ± 0.64	6.34 ± 0.41	***
Sr	7.93 ± 1.20	4.86 ± 0.53	*
Ag #	0.98 ± 0.08	0.64 ± 0.02	***
Cs #	17.3 ± 1.40	9.47 ± 0.88	***
Ba	6.06 ± 0.74	4.09 ± 0.39	*
Pb	4.28 ± 0.29	2.85 ± 0.11	***
U #	115.8 ± 15.8	74.2 ± 11.0	*
Σ hMT-inducers ^Δ^	109.9 ± 18.9	54.39 ± 3.25	**
Σ MT-binding metals ^ΔΔ^	2.33 ± 0.05	2.13 ± 0.03	**

Data (by ICPMS) of endemic control from Padaviya ^1^ and nonendemic control from Padalangala ^2^ were compared by Student’s *t*-test following log10 transformation (* *p* < 0.05, ** *p* < 0.01, and *** *p* < 0.001), and presented as mean ± SEM; Be, Cr, Se, Cd and Ti were at *p* > 0.05 (data not shown) #, in ng/mg protein. Σ hMT, total human metallothionein; ^Δ^, included Cd, Zn, Cu, As, Pb, Ni, and Cr; ^ΔΔ^, included Cu, Ag, Zn, Cd, Co, Ni, Fe, Pb, and Ag.

## Data Availability

Data utilized in the paper can be made available from the corresponding author for purposes agreed upon.
